# Effect of Fresnel prism in small-angle esotropia (≤ 20 prism diopters) with fixation preference

**DOI:** 10.1007/s00417-024-06662-z

**Published:** 2024-10-15

**Authors:** Hye Jun Joo, Seong-Joon Kim

**Affiliations:** 1 Department of Ophthalmology, Seoul National University Hospital and College of Medicine, 101, Daehak-Ro Jongno-Gu, Seoul, South Korea 03080; 2 Seoul National University College of Medicine, Seoul, South Korea

**Keywords:** Small-angle esotropia, Amblyopia, Fresnel prism, Non-surgical treatment

## Abstract

**Purpose:**

We aimed to investigate the effects of Fresnel prism treatment for small-angle esotropia (≤ 20 prism diopters [PD]) with fixation preference.

**Methods:**

We included 32 patients with remaining esotropia ≤ 20 PD measured using the simultaneous prism and cover test (SPCT) after full refractive error correction. Fresnel prism was applied to make patients orthotropic with glasses. Treatment was discontinued if remaining esotropia ≤ 4 PD was sustained during two consecutive follow-ups (2-month intervals) or if the angle continued to increase with prism adaptation. Patients were divided into treatment success and failure groups. Treatment success was defined by motor and visual acuity (VA) aspects. Criteria for motor success was residual esotropia ≤ 8 PD in patients with initial esotropia > 8 PD and a 30% decrease of esotropia in those with initial esotropia ≤ 8 PD. VA success was improvement of > 0.2 logMAR in the non-dominant eye.

**Results:**

The initial esodeviation angle was 6.92 ± 4.66 PD at distance and 10.53 ± 5.58 at near. The logMAR VA was 0.10 ± 0.13 and 0.26 ± 0.20 in the dominant and non-dominant eye, respectively. Among the 32 patients, 14 showed motor success. Among 26 patients whose VA could be measured, 15 showed VA success. Factors influencing motor success were a small amount of maximum prescribed Fresnel prism, less frequent need for Fresnel prism adaptation, and high percentage of achieving orthotropia with Fresnel prism treatment. VA success was influenced by low frequency of anisometropia and the small amount of maximum prescribed Fresnel prism.

**Conclusion:**

Fresnel prism could be a non-invasive treatment option for some patients with small-angle esotropia with fixation preference.

**Key messages:**

***What is known***

- The optimal approach for addressing small-angle esotropia is a topic of debate.
- Not much research has been conducted on Fresnel prism treatment in patients with small-angle esotropia.

***What is new***

- Motor success and visual acuity improvement were observed in some patients undergoing Fresnel prism treatment.
- Factors contributing to motor success were the small maximum angle of esodeviation, the less frequent necessity for Fresnel prism adaptation, and the achievement of orthotropia during Fresnel prism treatment.
- Visual acuity improvement was hindered by the presence of anisometropia and the large maximum prescribed amount of Fresnel prism.
- Fresnel prism treatment can be used as a treatment option for some patients with small-angle esotropia with fixation preference.

## Introduction

The optimal treatment for managing small-angle esotropia is under debate. The first step during a basic approach to managing esotropia is a full correction of the hypermetropic refractive error. Then, various treatment options can be considered, such as occlusion, prism, miotics, botulinum toxin injection, and surgical intervention, for any remaining esodeviation [1–3]. When the residual angle falls within the marginal range for surgical intervention, it is uncertain whether invasive treatment should be chosen, given the potential complications [4].

Previous studies by the authors have shown that in patients with accommodative esotropia, reducing the deviation angle to within 4 prism diopters (PD) is necessary to achieve true stereopsis or bifoveal fixation [5]. Additionally, in patients with slightly larger esodeviation, showing a feature of monofixation syndrome, the authors have observed that reducing the angle with a Fresnel prism improves both visual acuity (VA) and stereopsis [6].

Building on these previous studies, we aimed to assess whether these treatments were also effective in reducing the deviation angle. If successful, such non-invasive approaches could offer valuable options for managing small-angle esotropia, especially in cases where the optimal treatment strategy is unclear. Consequently, we sought to evaluate the effectiveness of Fresnel prism therapy for small-angle esotropia and identify the characteristics of patients who respond well to this treatment.

## Methods

This study adhered to the Declaration of Helsinki, and the requirement for informed consent was waived by the Institutional Review Board of Seoul National University Hospital (2312–075-1492) owing to the study’s retrospective design.

### Participants

We performed a retrospective chart review of children who visited the Department of Pediatric Ophthalmology at the Seoul National University Children’s Hospital in South Korea between 2010 and 2023. Participants were drawn from the patient load from a single surgeon (SJK). Children were included in this study if they met the following criteria: (1) remaining esotropia of ≤ 20 prism diopters (PD) as measured by the simultaneous prism and cover test (SPCT) after adaptation to full refractive error correction; and (2) had a fixation preference in one eye. Patients with a history of strabismus surgery were also included in the study. Patients with paralytic or restrictive esotropia; presence of other ophthalmic diseases such as corneal opacity, cataract, glaucoma, or optic nerve disease; poor cooperation during the prism cover test; and presence of a systemic anomaly such as a neurological disorder or developmental delay were excluded.

### Ophthalmic examination [6]

Cycloplegia was achieved by administering one drop of 1% cyclopentolate three times at intervals of 5 min; cycloplegic retinoscopy was performed 30 min after the last administration when the pupillary light reflex was eliminated. Full correction of astigmatism and anisometropia was generally implemented, and compliance with wearing glasses for more than 80% of the day was confirmed at each visit through reports from the parents. Patients were followed up after 2 months, and if esotropia smaller than 10 PD persisted after full spectacle correction of hyperopia, cycloplegic refraction was performed again using atropine twice daily for 3 days. The VA was measured using a Snellen chart in a fixed place under the same environmental conditions, including distance and illuminance, and converted into the logarithm of the minimum angle of resolution (logMAR) for statistical analysis.

Fixation preference for the binocular fixation pattern was evaluated by covering the child’s dominant fixating eye, forcing the deviating eye to fixate. After the fixation of the deviating eye stabilized, the cover was removed, and the fixating eye was observed for the duration required to resume fixation. Anisometropia was defined as a spherical equivalent difference of more than 1.5 diopters (D) between the two eyes. Amblyopia was defined as two or more lines of interocular differences in the Snellen VA. Patients with a difference of two lines or more in VA in each eye were considered to have amblyopia and primarily treated with part-time [7, 8] or full-time occlusion treatment [9] along with spectacle-wearing. Fresnel prism treatment was considered when there was no improvement in vision after at least six months of occlusion treatment.

The angle of deviation was determined by performing a simultaneous prism cover test, which was performed by an experienced physician (SJK). We analyzed the angle of tropia instead of the angle of phoria. If there was a disparity between near and distance deviation angles, Fresnel prisms were prescribed based on the larger value. Sensory fusion was tested using the Worth-4-Dot test near (0.33 m) and at a distance (6 m). Stereoacuity was measured using the Titmus stereo test (Stereo Optical, Chicago, IL, USA). If the patient had no measurable stereoacuity where the largest disparity could not be passed, a value of 6000 arcsecs was assigned to nil stereopsis for statistical analysis. All values were transformed to log arcsec for the analysis [5, 10]. We analyzed the results before and after management using a Fresnel prism.

### Management with Fresnel prism

Base-out Fresnel press-on prisms (3 M Press-On Optics; 3 M Health Care, St Paul, Minnesota, USA) were applied to the posterior surface of the glasses over the dominant eye. The prism was prescribed according to the angle of esodeviation to make the patient orthotropic after wearing glasses with a Fresnel prism [6]. The first follow-up was performed 2 months later, and more prisms were added if patients presented with remained esotropia after prismatic correction on follow-up visits. The Fresnel prism was discontinued when the patient consistently exhibited esodeviation ≤ 4 PD [5] during two consecutive visits with the previously prescribed Fresnel prism. Alternatively, if there was a continuous increase in esodeviation with prism adaptation, the procedure was discontinued.

### Group classification

Patients were categorized into two groups based on treatment success, which was assessed based on both motor and VA aspects. Motor success was defined as having residual esotropia ≤ 8 PD after discontinuing Fresnel prism in patients with initial esotropia > 8 PD and a 30% decrease of esotropia in those with initial esotropia ≤ 8 PD. VA success was defined as VA improvement of more than 0.2 logMAR in the non-dominant eye.

### Main outcome measures

The following parameters were reviewed before and after Fresnel prism treatment: age, sex, refractive error, VA, presence of amblyopia, angle of deviation, and stereoacuity. Subgroup analysis was performed according to the final motor and VA outcomes.

### Statistical analysis

All statistical analyses were performed using SPSS software (version 27.0; IBM, Armonk, NY, USA). Fisher’s exact test was used to compare categorical variables between the two groups. The Mann–Whitney U test was used for continuous variables. Statistical significance was set at *p* values < 0.05.

## Results

A total of 32 patients were included in this study (Table 1). The mean age of the patients at the first visit was 3.0 ± 1.4 years. Twenty-seven patients were diagnosed with accommodative esotropia and 5 with non-accommodative esotropia. Four patients underwent strabismus surgery before Fresnel prism treatment (two underwent BMR recession for partial accommodative esotropia and two BMR recession for infantile esotropia).

**Table 1:** Demographics and clinical characteristics of the patients

Variables	*N* = 32
Diagnosis (accommodative ET:non-accommodative ET)	27:5
Sex (male:female)	10:22
Age of onset (years)	2.2 ± 1.3 (0.3–5)
Age of first hospital visit (years)	3.0 ± 1.4 (0.3–6.5)
Age at prescription of FP (years)	4.9 ± 1.9 (1–11)
Cycloplegic refractive errors (D)	
Dominant eye	3.06 ± 2.42 (− 1.00 to + 10.25)
Non-dominant eye	3.77 ± 2.43 (− 1.50 to + 11.00 D)
VA (logMAR)	N = 26
Dominant eye	0.10 ± 0.13 (− 0.08 to + 0.40)
Non-dominant eye	0.26 ± 0.20 (0.00 to + 0.70)
Anisometropia (n, %)	5/32 (15.6%)
Amblyopia (n, %)	20/26 (76.9%)
Initial angle of esodeviation	
Distance	6.92 ± 4.66 (0–20)
Near	10.53 ± 5.58 (4–20)
Initial stereoacuities (log arcsec) (n = 12)	3.08 ± 0.76
Duration of FP (months)	10.47 ± 6.60 (4–28)

*D* diopter, *ET* esotropia, *VA* visual acuity, *FP* Fresnel prism, amblyopia, two or more lines of interocular difference in Snellen *VA*, anisometropia, spherical equivalent difference of more than 1.5 diopters (D) between the two eyes

Values are presented as mean ± standard deviation (range) or number (%)

### Changes in VA and motor alignment after Fresnel prism use

Out of the total 32 patients included in the study, VA measurements were available for 26 patients before and after treatment, and measurement of angle of deviation were available for all 32 patients. After management using the Fresnel prism, a significant improvement was observed in the mean VA of the non-dominant eye, from 0.26 ± 0.20 logMAR to 0.20 ± 0.16 logMAR (*p* = 0.010). No significant changes were observed in the VA of the dominant eye. The mean initial angle of esodeviation was 6.92 ± 4.66 PD at distance and 10.53 ± 5.58 PD at near. After discontinuing the Fresnel prism, the mean angle of deviation was 9.02 ± 10.07 PD at distance and 10.53 ± 12.27 PD at near (*p* = 0.333 and 0.885, respectively) (Table 2). Table 3 presents the presence or absence of improvement in VA and the angle of esodeviation in individuals who were able to measure both VA and esodeviation angle.

**Table 2:** Changes in sensory and motor status after management using Fresnel prism

Variables	Before	After	*p*-value*
VA (logMAR) (n = 26)			
Dominant eye	0.10 ± 0.13	0.10 ± 0.14	0.242
Non-dominant eye	**0.26 ± 0.20**	**0.20 ± 0.16**	**0.010**
Angle of esodeviation (n = 32)			
Distance	6.92 ± 4.66	9.02 ± 10.07	0.333
Near	10.53 ± 5.59	10.53 ± 12.27	0.885
Interocular difference (N-D)	3.61 ± 6.15	1.50 ± 4.22	0.088
Stereoacuities (log arcsec) (n = 12)	3.08 ± 0.76	2.90 ± 0.84	0.279

*VA *visual acuity, *N *near, *D *distance

Boldface values denote statistically significant results

^*^Wilcoxon signed rank test

**Table 3:** Number of individuals showing changes in esodeviation and visual acuity during Fresnel prism treatment

	VA improvement ( +)	VA improvement ( −)
Angle improvement ( +)	**7**	**5**
Angle improvement ( −)	**8**	**6**

*D* diopter, *VA* visual acuity

VA improvement is defined as VA improvement of > 0.2 logMAR in the non-dominant eye; angle improvement is defined as residual esotropia (SPCT) ≤ 8 PD in patients with initial esotropia > 8 PD and 30% decrease of esotropia in patients with initial esotropia ≤ 8 PD

### Subgroup analysis according to motor outcome after Fresnel prism treatment

Some patients showed significant improvement in motor alignment after discontinuing Fresnel prism treatment. Accordingly, a subgroup analysis was conducted based on the success of motor outcomes (Table 4). Fourteen patients demonstrated treatment success, whereas 18 showed treatment failure. Among various factors, the maximum prescribed amount of the Fresnel prism (16.07 ± 8.43, 21.78 ± 6.22, *p* = 0.011), the maximum angle of esodeviation at distance (15.79 ± 5.39, 24.11 ± 7.79, *p* = 0.004) and near (17.93 ± 7.30, 25.06 ± 7.20, *p* = 0.009), the percentage of frequent prism adaptation need (5/14, 16/18, *p* = 0.013), and the percentage of achieving orthotropia and ≤ 4 esotropia during Fresnel prism treatment showed significant differences. During Fresnel prism treatment, some patients exhibited an increase in esodeviation with prism adaptation, resulting in a maximum prescribed Fresnel prism amount exceeding 20 PD for certain patients. The angle measured by the alternate prism cover test (APCT) before Fresnel prism treatment showed no significant correlation with the maximum angle of deviation after prism adaptation (Spearman correlation analysis, *p* = *0.163*). Furthermore, patients with larger APCT values, indicating greater latent deviation, did not experience worse treatment outcomes (Mann–Whitney U test, *p* = *0.825*).

**Table 4:** Subgroup analysis according to final motor outcome

	Success (*n* = 14)	Failure (*n* = 18)	*p*-value
Sex (male:female)	5:9	5:13	0.459*
Age of onset (years)	2.2 ± 1.2	2.3 ± 1.5	0.985†
Age of first hospital visit (years)	2.9 ± 1.3	3.0 ± 1.6	0.896†
Onset to hospital visit (months)	9.5 ± 14.2	8.8 ± 13.2	0.925†
Cycloplegic refractive errors (D)			
Dominant eye	2.90 ± 1.86	3.20 ± 2.83	0.896†
Non-dominant eye	3.43 ± 1.87	4.03 ± 2.81	0.722†
Glasses prescription (months)	17.0 ± 16.3	11.20 ± 16.3	0.319†
VA (logMAR)	N = 12	N = 14	
Dominant eye	0.10 ± 0.14	0.09 ± 0.12	0.980†
Non-dominant eye	0.20 ± 0.12	0.32 ± 0.23	0.131†
Anisometropia (n, %)	2/17	3/15	0.537*
Amblyopia (n, %)	8/12 (%)	12/14 (83.3%)	0.248*
Patching (n, %)	5/14	5/18	0.459*
Initial angle of esodeviation (PD)			
Distance	6.79 ± 6.20	7.03 ± 3.19	0.722†
Near	12.00 ± 5.42	9.39 ± 5.59	0.145†
Interocular difference (N-D)	5.21 ± 6.86	2.36 ± 5.40	0.145†
Age at prescription of FP (years)	5.2 ± 2.4	5.0 ± 2.0	0.925†
Initially prescribed FP (PD)	12.71 ± 4.53	12.78 ± 4.95	0.808†
Maximum prescribed FP (PD)	**16.07 ± 8.43**	**21.78 ± 6.22**	**0.011†**
Maximum angle of esodeviation (PD)			
Distance	**15.79 ± 5.39**	**24.11 ± 7.79**	**0.004†**
Near	**17.93 ± 7.30**	**25.06 ± 7.20**	**0.009†**
Prism adaptation twice or more	**5/14**	**16/18**	0.003*
Orthotropia with Fresnel prism	**11/14**	**6/18**	0.013*
≤ 4PD ET with Fresnel prism	**13/14**	**11/18**	0.047*
Duration of FP (months)	12.07 ± 7.09	9.72 ± 9.11	0.107**†**
Fresnel removal–last FU (months)	16.79 ± 7.39	10.61 ± 12.09	0.054**†**
Age at final follow-up (years)	7.43 ± 2.62	6.78 ± 2.60	0.639**†**

*D* diopter, *PD* prism diopter, *ET* esotropia, *VA* visual acuity, *N *near, *D* distance, *FP* Fresnel prism

Values are presented as the mean ± standard deviation (range) or number (%)

^*^Fisher’s exact test, †Mann–Whitney U test

### Subgroup analysis according to visual acuity outcome after Fresnel prism treatment

Fifteen patients (58%) gained more than 0.2 logMAR in the non-dominant eye after treatment (Table 5). Among the various factors, the presence of anisometropia and the larger amount of maximum prescribed Fresnel prism were notably associated with the failure of vision improvement by 0.2 logMAR or more (*p* = 0.007 and 0.047, respectively). Before treatment, 20 patients were diagnosed with amblyopia, 12 of whom (60%) showed resolution of the condition, demonstrating the effectiveness of Fresnel prism treatment for amblyopia.

**Table 5:** Subgroup analysis according to sensory outcome (VA improvement of > 0.2 logMAR in the non-dominant eye)

	Success (*n* = 15)	Failure (*n* = 11)	*p*-value
Sex (male:female)	5:10	4:7	0.598*
Age of onset (years)	2.5 ± 1.4	2.6 ± 1.3	0.855†
Age of first hospital visit (years)	3.3 ± 1.5	3.2 ± 1.3	0.979†
Onset to hospital visit (months)	9.1 ± 14.5	9.0 ± 15.1	0.613†
Cycloplegic refractive errors (D)			
Dominant eye	2.53 ± 1.75	2.45 ± 2.12	0.838†
Non-dominant eye	2.98 ± 1.65	3.84 ± 2.22	0.198†
Glasses prescription (months)	14.2 ± 16.7	13.5 ± 19.4	0.683†
VA (logMAR)			
Dominant eye	0.11 ± 0.13	0.08 ± 0.12	0.646†
Non-dominant eye	0.24 ± 0.19	0.30 ± 0.21	0.443†
Anisometropia (n, %)	**0/15 (0%)**	**5/11 (45.5%)**	0.007*
Amblyopia (n, %)	12/15 (80%)	8/11 (72.7%)	0.509*
Patching (n, %)	5/15	3/11	0.543*
Initial angle of esodeviation			
Distance	7.30 ± 3.54	4.73 ± 4.13	0.121†
Near	9.64 ± 4.96	9.64 ± 5.71	0.760†
Interocular difference (N-D)	2.33 ± 4.24	4.91 ± 7.40	0.474†
Age at prescription of FP (years)	5.7 ± 1.3	5.9 ± 2.1	0.838†
Initially prescribed FP (PD)	12.20 ± 5.03	11.82 ± 3.71	0.919†
Maximum prescribed FP (PD)	**15.80 ± 5.23**	**22.73 ± 9.36**	**0.047†**
Maximum angle of esodeviation (PD)			
Distance	18.27 ± 7.18	20.64 ± 8.48	0.474†
Near	19.20 ± 6.90	23.64 ± 8.94	0.148†
Prism adaptation twice or more	8/15	9/11	0.138*
Orthotropia with Fresnel prism	9/15	7/11	0.588*
≤ 4PD ET with Fresnel prism	14/15	8/11	0.188*
Duration of FP (months)	10.20 ± 6.67	12.27 ± 10.89	0.721**†**
Fresnel removal–last FU (months)	15.93 ± 9.59	12.55 ± 12.68	0.330**†**
Age at final follow-up (years)	7.8 ± 1.6	8.0 ± 2.8	0.760**†**

*D* diopter, *PD* prism diopter, *ET* esotropia, *VA* visual acuity, *N* near, *D* distance, *FP* Fresnel prism

Values are presented as mean ± standard deviation or number (%)

^*^Fisher’s exact test, †Mann–Whitney U test

### Motor outcomes following discontinuation of Fresnel prism treatment

**Fig. 1:**
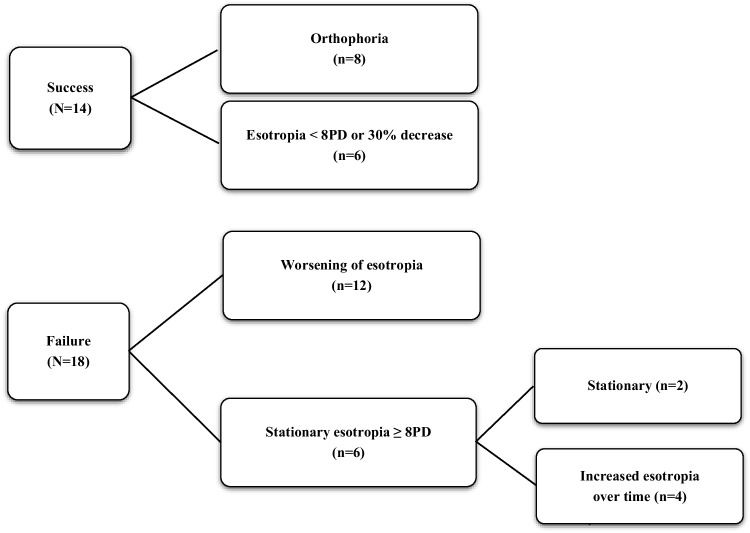
Motor outcomes following discontinuation of Fresnel prism treatment

The motor outcomes following discontinuation of the Fresnel prism treatment are depicted in Fig. 1. The average follow-up period after discontinuing Fresnel prism treatment (excluding those who underwent surgery right after discontinuing Fresnel prism treatment) was 19.5 months (ranging from 6 to 39 months). Out of the 32 patients, 14 showed improvement in strabismus angle (initially ranging from 4 to 20 PD), with all maintaining this improvement without worsening by the final follow-up. Twelve experienced worsening of their strabismus angle (initially ranging from 4 to 20PD). 6 showed unchanged esodeviation pre- and post- treatment, with 4 showing worsening of the esodeviation over time and 2 remaining stable.

## Discussion

In this study, we demonstrated the effect of Fresnel prism treatment in patients with small-angle esotropia and fixation preference. Approximately 44% of the patients showed motor success. Factors related to motor success indicated that a smaller angle after prism adaptation was a prognostic factor of success. Approximately 58% of patients showed VA success, defined as a VA gain of more than 0.2 logMAR in the non-dominant eye. Failure to improve VA was observed in patients with larger angle after prism adaptation and anisometropia.

This study is significant because, first, it contributes to the management of small-angle esotropia, a condition for which the treatment options are typically limited. Traditionally, surgery has been the primary approach [11], with occlusion therapy occasionally used as an initial intervention before surgical consideration [12]. However, our study demonstrated the efficacy of the Fresnel prism treatment in reducing the angle of deviation in some patients with small esotropia. The use of a base-out Fresnel prism helped regain binocular fusion, which may have strengthened the fusional vergence system, as reported in previous studies [3, 13, 14]. Although not universally effective, the observed benefits in a subset of cases indicate that it should be considered as a viable therapeutic option. Of course, children lacking the ability of binocular vision (BV) would not respond to the treatment and may experience worsening of strabismus over time. However, we believe that deterioration in these children would have occurred regardless of the treatment. Moreover, in young children, accurately determining the presence or absence of BV based on a few attempts of stereoacuity tests is challenging. Thus, concluding the absence of BV from limited tests may not always be accurate. Therefore, attempting such treatment in cases where BV is uncertain may still be worthwhile.

Second, the Fresnel prism treatment is significant for the treatment of amblyopia. The mechanisms underlying amblyopia can be categorized into two main types [15]: those resulting from vision deprivation during critical periods of visual immaturity and others arising from abnormal binocular interaction leading to impaired visual development. The conventional mainstay of amblyopia treatment is patching the normal fellow eye to improve the monocular function of the amblyopic eye [16]. Although this approach can reduce the imbalance in signals entering the eyes, it does not address the issue of “abnormal binocular interaction” in amblyopia. Recently, there has been an emphasis on amblyopia treatment focusing on binocularity-stimulating treatments using virtual reality technology. This approach encourages both eyes to work simultaneously by providing a strong stimulus to the amblyopic eye and a weak one to the normal sound eye, leading to cortical alterations [17, 18]. The Fresnel prisms are made of a thin plastic sheet comprising multiple angular grooves on one side [19]. Such an effect not only corrects ocular alignment but also creates a blurred image, leading to a decrease in the VA in the eye wearing the prism [20]. When applied to the dominant eye, it can inhibit visual input to the dominant eye and contribute to the balance between both eyes [21]. Our study findings indicate that using the Fresnel prism to improve binocular interaction can aid in encouraging the simultaneous utilization of both eyes, potentially achieving a binocularity-stimulating treatment effect.

Prisms have been used in the management of esotropia under various conditions. Prism adaptation using a prism may help determine the amount of surgery required to prevent under-correction [22, 23]. The use of base-out prisms may help regain binocular fusion, stimulate fusional divergence, achieve a reduction in prismatic strength, and gain orthophoria [3, 13, 14]. Choe et al. [3] reported the motor outcome of prism glasses in partially accommodative esotropia with a residual esotropia of ≤ 20 PD and 7.3% of the patients were eventually weaned off prism glasses. However, prism glasses were symmetrically prescribed in both eyes to remove residual esotropia, and changes in VA following prism treatment were not investigated. In contrast, our study uniquely prescribed the Fresnel prism only to the dominant eye, and some patients demonstrated improvements in VA with or without changes in motor alignment. The Fresnel prism may have served as a form of amblyopia treatment, and enhancements in VA might have been affected by the motor alignment induced by the Fresnel prism. Our purpose in using the Fresnel prism in the dominant eye was to ensure symmetrical visual input in both eyes. This approach emphasizes the stimulation of binocular vision to alleviate the underutilization of the non-dominant eye, highlighting the unique strength of the Fresnel prism treatment.

An earlier investigation involving the application of Fresnel prisms in patients with esotropia < 8 PD and subnormal stereopsis showed improvements in the VA and stereopsis in some cases [6]. No changes were observed in the esodeviation angle after the Fresnel prism treatment. The explanation was that temporary changes in motor alignment caused by the Fresnel prism were not sufficient to form new neural connections, and the tight cortical connections in patients with monofixation syndrome cannot be broken. However, in our study, during a mean follow-up period of 19.5 months after discontinuing the Fresnel prism treatment, satisfactory motor alignment was maintained in some cases. The angle measured by the APCT before Fresnel prism treatment showed no significant correlation with the maximum angle of deviation after prism adaptation, suggesting that a larger latent deviation does not necessarily lead to a larger angle after prism adaptation (Spearman correlation analysis, *p* = *0.163*), nor was it linked to motor success (Mann–Whitney U test, *p* = *0.825*). Similarly, regarding VA outcomes, having amblyopia before treatment (Fisher’s exact test*, p* = *0.509*) or poor VA in the non-dominant eye (Mann–Whitney U test, *p* = *0.443*) did not result in poor VA outcomes. We believe that the firmness of monofixation status may play a crucial role in determining the success of treatment. For patients with strong neural connections to the noncorresponding areas, structural changes (new neural connections)—such as improvement in amblyopia or reduction in the angle of deviation—may have been more difficult because of the challenge of forming new synapses. This study focused on the Fresnel prism treatment in patients with small-angle esotropia and a fixation preference. Fresnel prisms were applied to the dominant eye, gradually adding up until achieving esotropia of ≤ 4 PD, to promote proper binocular vision. The decision to use the 4 PD criterion was based on a previous study [5]. The maximum angle of deviation for expected true stereopsis in refractive accommodative esotropia was ≤ 4 PD at distance and ≤ 5 PD at near [5], while the neuroanatomical findings of Wong et al. suggested that the true stereopsis may be possible only with a misalignment of ≤ 4 PD esotropia [24]. A notable strength of our study using the Fresnel prism is that it helps maintain motor alignment, potentially reducing the esodeviation after treatment cessation, while also functioning as an occlusion therapy for the dominant eye. This dual role minimizes the disparity between the eyes and promotes binocular visual function.

Our study has several limitations. First, the absence of control groups and the varied clinical diagnoses and treatment trajectories pose significant challenges. Second, due to the retrospective nature of this study, there was variability in the time intervals between the onset of the condition, presentation, surgery or occlusion, and the initial prescription of Fresnel prism among patients. Additionally, patients who were unable to undergo stereoacuity tests due to young age and poor cooperation were included in the study. No significant differences were observed when comparing treatment outcomes in patients in whom sensory tests were available. This result can be attributed to the inclusion of only fully cooperative patients with good stereoacuity. The inability to conduct such evaluations and the inclusion of a heterogeneous patients are limitations of our study. Future larger-scale prospective research in a more controlled setting, incorporating a range of tests to assess binocular vision, is essential.

In conclusion, our study demonstrates that Fresnel prisms can serve as a viable treatment option for some individuals with small-angle esotropia and amblyopia. While certain studies have revealed that a deep-seated monofixation status may impede the effectiveness of Fresnel prism treatment, it remains a potential therapeutic option for some patients with small-angle esotropia and amblyopia.
